# Factors associated with glycaemic control and diabetes complications in patients at Bugando Medical Centre, Mwanza, Tanzania: A cross-sectional study design

**DOI:** 10.1371/journal.pone.0308659

**Published:** 2024-08-30

**Authors:** Aneth H. Muchunguzi, Emmanuel Kimaro, Eveline T. Konje, Benson R. Kidenya, Amani T. Mori, Eliangiringa Kaale

**Affiliations:** 1 Department of Pharmaceutics, School of Pharmacy, Catholic University of Health and Allied Sciences (CUHAS), Mwanza, Tanzania; 2 Department of Biostatistics and Epidemiology, School of Public Health, Catholic University of Health and Allied Sciences (CUHAS), Mwanza, Tanzania; 3 Department of Biochemistry, School of Medicine, Catholic University of Health and Allied Sciences (CUHAS), Mwanza, Tanzania; 4 Department of Global Public Health and Primary Care, Section for Ethics and Health Economics, University of Bergen, Bergen, Norway; 5 Pharm R&D Laboratory, School of Pharmacy, Muhimbili University of Health and Allied Sciences (MUHAS), Dar es Salaam, Tanzania; 6 Department of Medicinal Chemistry, Muhimbili University of Health and Allied Sciences (MUHAS), Dar es Salaam, Tanzania; Bangladesh University of Health Sciences, BANGLADESH

## Abstract

**Background:**

Glycaemic control is essential for improving the quality of life in patients with Diabetes Mellitus (DM). Untreated hyperglycaemia can result in numerous severe and life-threatening complications, such as damage to the eyes, kidneys, nerves, heart, and peripheral vascular system. Appropriate glycaemic control and management is fundamental to prevent and delay diabetes complications. Therefore, this study aims to assess the prevalence of poor glycaemic control, its associated factors, and the prevalence of diabetes-related complications among DM patients.

**Methods:**

A cross-sectional study was conducted among 340 DM patients treated at Bugando Medical Center from 4^th^ - 30^th^ April 2023 to determine the prevalence of poor glycaemic control and its predictors. Secondary data from 7952 DM patients treated between April 2022 and 30^th^ May 2023 were used to determine DM-related complications. STATA 15 version …was used for analysis.

**Results:**

Out of 340 patients, 66.4% had poor glycaemic control with HbA_1c_ or Random Blood Glucose greater than 7% or 7mmol/L, respectively. Older age, duration of DM of more than 10 years, insulin therapy, and those unaware of glycaemic target goals were factors associated with poor glycaemic control. (AOR: 2.46, 95% CI: 1.28–6.01, P = 0.03), (AOR: 3.15, 95% CI: 2.22–6.55, P = 0.016), (AOR: 3.07, 95% CI: 2.10–6.12, P = 0.022) and (AOR: 3.42, 95% CI: 2.17–5.97, P = 0.001), **respectively. Of the 7952 patient records reviewed indicated that 44.5% had complications, of which 25.8% had neurological complications and 55.3% had multiple complications.**

**Conclusion:**

Two-third of DM patients failed to achieve good glycaemic control and about half of the patient’s records reviewed indicated they developed diabetic complications. Thus appropriate interventions are necessary to improve glycaemic control and prevent or control complications among DM patients.

## Introduction

Glycaemic control is crucial for enhancing the quality of life in patients with Diabetes Mellitus (DM) [**1**]. When hyperglycaemia is left untreated, it can lead to many serious life-threatening complications, including damage to the eye, kidneys, nerves, heart, and peripheral vascular system [2]. Approximately 537 million adults are living with diabetes, and more than 1.2 million children and adolescents are living with type 1 diabetes. About 24 million adults aged between 20–79 years are living with diabetes in Africa [2]. The total number of people living with diabetes is projected to rise to 643 million by 2030 and to 783 million by 2045 [2].

Appropriate glycaemic control and management are fundamental to preventing and delaying diabetes complications. Poor glycaemic control (HbA1c greater than 7% and Random Blood Glucose (RBG) greater than 7 mmol/L) is highly correlated with a high burden of diabetes complications, including microvascular and macrovascular complications that result in damage to the eyes, kidneys, nerves, heart, and peripheral vascular system [3]. To assess the burden of diabetes complications, the current study focused on DM patients who attended the diabetes clinic at Bugando Medical Center (BMC) in Tanzania over one year, from April 2022 to March 2023.

Additionally, not all patients with diabetes can achieve adequate glycaemic control due to various factors such as lack of access to healthcare, financial constraints, age, sex, educational status, body mass index (BMI), family history, duration of diabetes, lifestyle practices (such as diet, smoking, and physical activity), and comorbid conditions (such as hypertension, dyslipidaemia, and kidney disease) [4]. Therefore, identifying the challenges and factors associated with poor glycaemic control is crucial for instituting appropriate interventions to improve glycaemic control and prevent target organ damage and other chronic complications arising from diabetes [5].

A few studies have been conducted in Tanzania to determine predictors associated with poor glycaemic control and the prevalence of diabetes complications. A study by Kamuhabwa *et al*. revealed that the majority (69.7%) of diabetes patients had poor glycaemic control [5]. Therefore, this study aims to assess the prevalence of poor glycaemic control, its associated factors, and the prevalence of diabetes-related complications among DM patients.

## Methods

### Study setting and design

This cross-sectional study was conducted at Bugando Medical Centre (BMC), a tertiary hospital located in Mwanza, Tanzania. BMC serves approximately 10,000 diabetic patients annually, with diabetic clinics held twice per week. The study was conducted from April 4 to 30, 2023, and incorporated secondary data from patient records who were treated at BMC from April 2022 to March 2023.

### Study population

The study included both type 1 and type 2 diabetes mellitus (DM) patients attending diabetic clinics at BMC. It comprised two distinct study populations: the first population consisted of diabetes patients treated at BMC from April 2023 to May 2023. The primary data from this sample was used to determine the prevalence of poor glycaemic control and the predictors. The second population involved secondary data from all DM patients who were treated at BMC from April 2022 to March 2023, which was used to determine the proportion of diabetes complications. For the first sample, only Type 1 and type 2 DM patients attending Bugando Medical Centre who consented to participate were included, while severely ill individuals, newly diagnosed patients, and pregnant women were excluded from the study.

### Sample size and sampling technique

The required sample size for the first study was 325 diabetes patients, determined using Kish Leslie’s formula [6]. To achieve this, participants were recruited serially until the required sample size was reached for the first study population. Participants were selected based on accessibility and willingness to participate rather than through random selection. For the second study population, a total of 7952 patient data from their medical records were extracted.

### A formula for Kish Leslie

$$\text{n}={\text{Z}}^{2}*\text{P}(1-\text{P})/{\left(\varepsilon \right)}^{2}$$

$$\text{n}=1.96^{2*}0.697(1-0.697)/{\left(0.05\right)}^{2}$$

n=325

Estimated sample size, n = 325 (but we have interviewed only 340 during the study period due to the availability of the study participants)

Where:

p-proportion of patients expected to have DM complications [5]

n- Estimated sample size

ε- Level of significance is 0.05

### Data collection

Data were obtained retrospectively from patients’ medical records from April 1, 2022, to March 31, 2023, using a checklist to determine the proportion of diabetes complications among the second group of DM patients (7952) attending BMC. A structured questionnaire, prepared in both English and Swahili languages, was used to determine predictors associated with poor glycaemic control and clinical outcomes (HbA1c and RBG) among the first group of DM patients (340). Clinical outcomes data (HbA1c and RBG) were also recorded from medical files.

### Data analysis

The data, initially cleaned using Microsoft Excel 2013, underwent analysis in STATA 15. Chi-square tests were employed to assess associations between variables and glycaemic control, defined as HbA1c < 7% or RBG < 7 mmol/L for adequate control, and HbA1c > 7% or RBG > 7 mmol/L for poor control, according to ADA guidelines. Regression modelling was then conducted to further explore predictors of glycaemic control, including age, insulin therapy, self-glucose monitoring, gender, BMI, and duration of diabetes. Logistic regression was used for binary outcomes. Findings were presented in tables, which included regression coefficients, confidence intervals, and p-values. Discussions highlighted the significance of identified predictors in diabetes management and their implications within the existing literature

### Ethics approval and consent to participate

This study received ethical approval from the CUHAS/BMC RESEARCH COMMITTEE (CREC), reference number (CREC/2541/2023). Informed consent was obtained from all individual participants included in the study. Participants were provided with comprehensive information about the study’s purpose, procedures, potential risks, and benefits, and their participation was entirely voluntary. Written consent forms were signed by each participant, ensuring their understanding and agreement to partake in the research.

## Results

### Socio-demographic characteristics of participants

A total of 340 diabetic patients were enrolled in this study, with 295 (87.8%) having type 2 DM and 45 (13.2%) having type 1 DM. The majority (54.1%) were aged between 45 and 64 years, and females constituted the majority at 187 (55%). Among the participants, 234 (68.8%) were urban residents, while 106 (31.2%) were rural residents. Additionally, 298 (87.6%) identified as Christians, 130 (38.2%) were retired, and 149 (43.8%) had attained secondary education (Table 1).

**Table 1:** Socio-demographic characteristics of participants.

Variables		All (n = 340)	Type 1 (n = 45)	Type 2 (n = 295)
Age	≤ 24	7 (2.1%)	7 (15.6%)	0 (0.0%)
	25–44	43 (12.6%)	31 (68.9%)	12 (4.1%)
	45–64	184 (54.1%)	7 (15.5%)	177 (60.0%)
	≥ 65	106 (31.2%)	0 (0.0%)	106 (35.9%)
Sex	Male	153 (45%)	21 (46.7%)	132 (44.7%)
	Female	187 (55%)	24 (53.3%)	163 (55.3%)
Residence	Rural	106 (31.2%)	12 (26.7%)	94 (31.9%)
	Urban	234 (68.8%)	33 (73.3%)	201 (68.1%)
Marital status	Single	23 (6.8%)	12 (26.7%)	11 (3.7%)
	Married	273 (80.3%)	31 (68.9%)	241 (81.7%)
	Widow/widower	44 (12.9%)	1 (2.2%)	43 (14.6%)
Religion	Christian	298 (87.6%)	39 (86.7%)	259(87.8%)
	Muslim	42 (12.4%)	6 (13.3%)	36 (12.2%)
Employment status	unemployed	13 (3.8%)	0 (0.0%)	13 (4.4%)
	Employed	80 (23.5%)	24 (53.3%)	56 (19.0%)
	Self employed	110 (32.3%)	13 (28.9%)	97 (32.9%)
	Student	7 (2.0%)	7 (15 6%)	0 (0.0%)
	retired	130 (38.2%)	1 (2.2%)	129 (43.7%)
Education status	No education	11 (3.2%)	0 (0.0%)	11 (3.7%)
	Primary	131 (38.5%)	9 (20.0%)	122 (41.4%)
	Secondary	149 (43.8%)	25 (55.6%)	124 (42.0%)
	Certificate	1 (0.3%)	0 (0.0%)	1 (0.3%)
	Diploma	10 (2.9%)	1 (2.2%)	9 (3.1%)
	Graduate	36 (10.6%)	10 (22.2%)	26 (8.8%)
	Postgraduate	2 (0.6%)	0 (0.0%)	2 (0.7%)

### Prevalence of poor glycaemic control among diabetic patients

**Fig 1:**
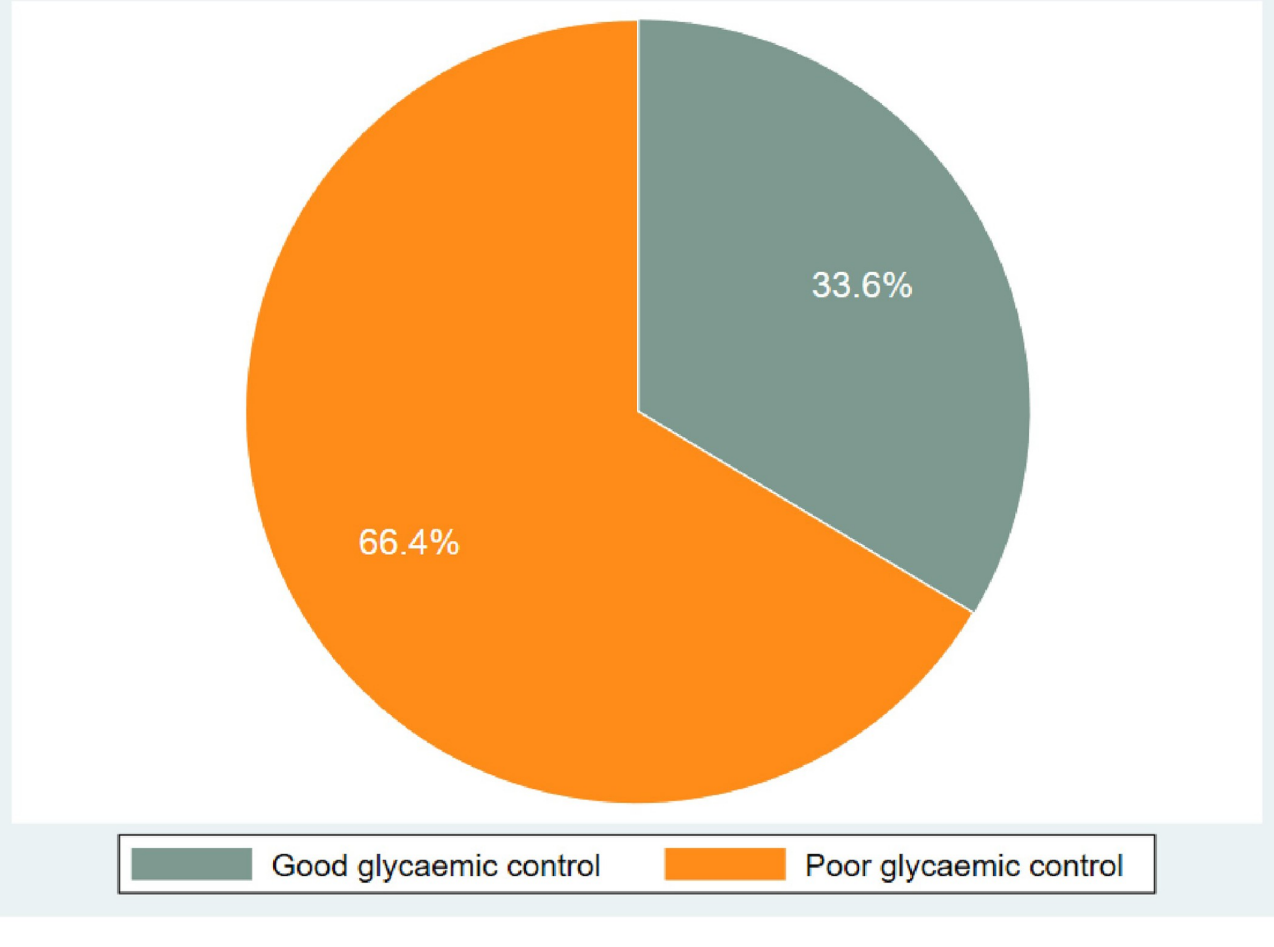
Proportion of glycaemic control among diabetic patients at BMC (n = 324).

Among the 340 study participants, 16 had no documented HbA1c and RBG values. The overall prevalence of poor glycaemic control was 66.4% (215/324) as shown in Fig 1. The proportion of poor glycaemic control was 37.8% among type 1 DM and 69.9% among type 2 DM. The prevalence of poor glycaemic control among the 45–64 age group was 119 (67.2%), among females was 118 (65.2%), among urban residents was 157 (69.5%), among the self-employed was 69.2%, among married individuals was 135 (70.2%), and among those with a primary level education was 73.0% as shown in Table 2.

**Table 2:** Glycaemic control among DM patients attending at BMC (n = 323).

Variables		Adequate glycaemic control (n = 109)	Poor glycaemic control (n = 214)
Age	≤ 24	4 (3.6%)	3 (1.4%)
	25–44	18 (16.5%)	18 (8.4%)
	45–64	58 (53.2%)	119 (55.6%)
	≥ 65	29 (26.6%)	74 (34.5%)
Sex	Male	46 (42.2%)	96 (44.9%)
	Female	63 (57.8%)	118 (55.1%)
Residence	Rural	40 (36.7%)	57 (26.6%)
	Urban	69 (63.3%)	157 (73.4%)
Marital status	Single	15 (13.8%)	7 (31.8%)
	Married	77 (70.6%)	181 (70.2%)
	Widow/widower	17 (15.6%)	26 (60.5%)
Employment status	unemployed	4 (3.7%)	9 (69.2%)
	Government employed	31 (28.4%)	41 (56.9%)
	Self employed	33 (30.3%)	74 (69.2%)
	Student	4 (3.7%)	3 (42.9%)
	retired	37 (33.9%)	87 (70.2%)
Education status	No education	4 (3.7%)	7 (3.3%)
	Primary	34 (31.2%)	92 (42.9%)
	Secondary	51 (46.7%)	88 (41.1%)
	Certificate	0 (0.0%)	1 (0.5%)
	Diploma	1 (0.9%)	9 (4.2%)
	Graduate	18 (16.5%)	16 (7.5%)
	Postgraduate	1 (0.9%)	1(0.5%)
Type of DM	Type 1	23 (21.1%)	14 (6.5%)
	Type 2	86 (78.9%)	200 (93.5%)

**Abbreviation:** DM, diabetes mellitus.

### Prevalence of diabetic complications among diabetic patients

**Fig 2:**
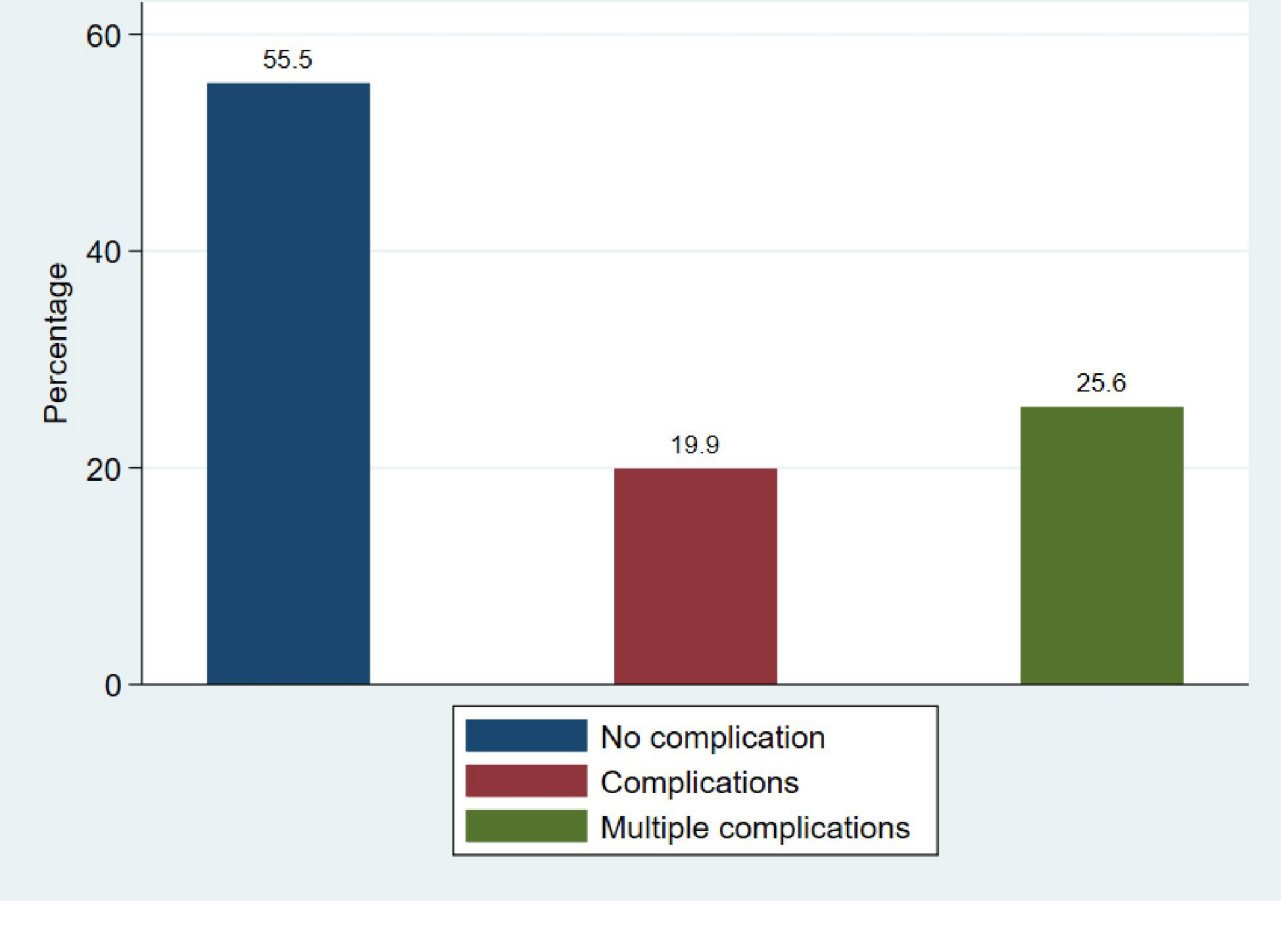
Proportion of diabetic complications at BMC (n = 324).

**Fig 3:**
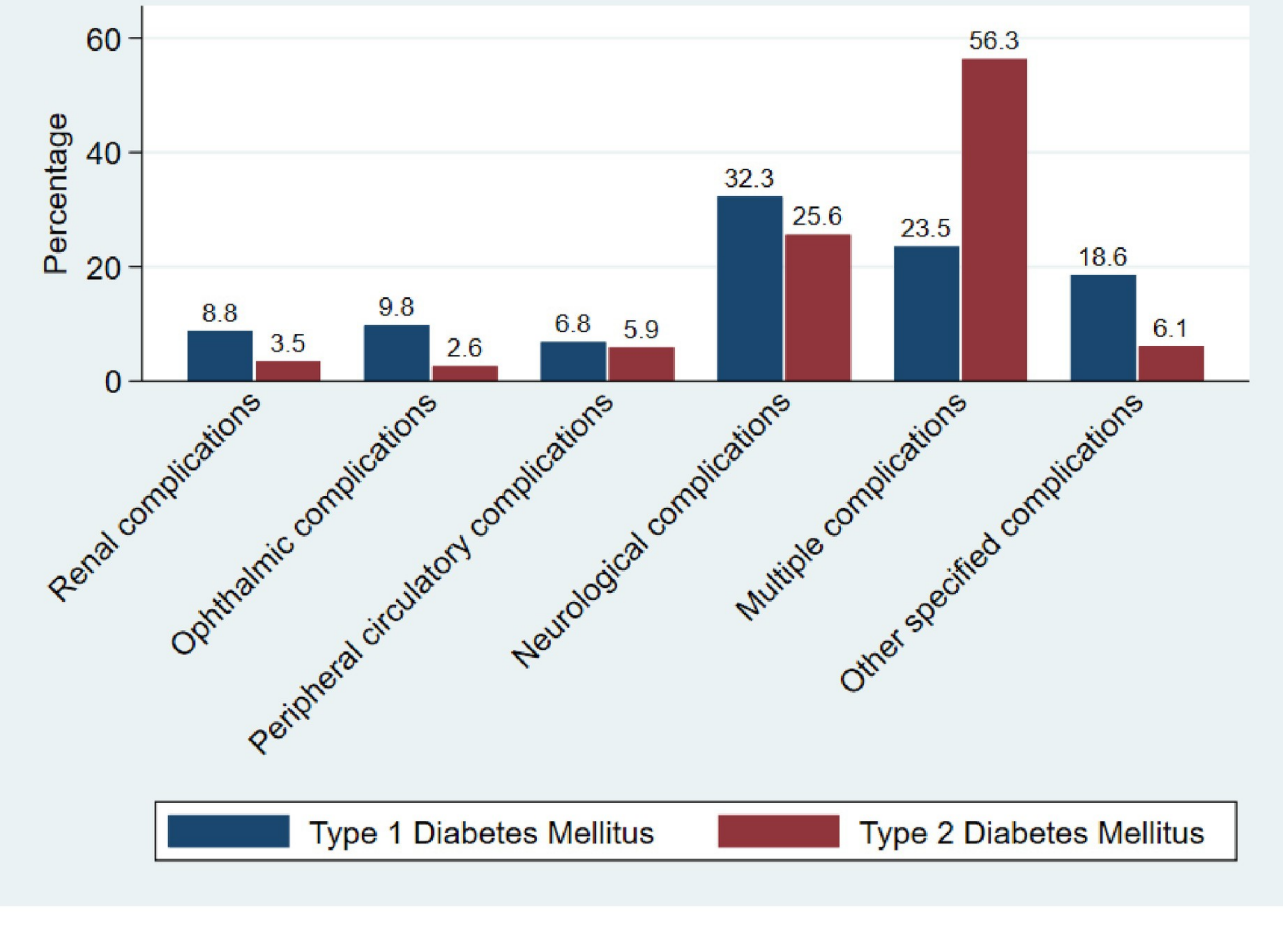
Diabetes complications by type at BMC (n = 3541).

The overall prevalence of diabetic complications was 44.5% among diabetic patients attending BMC, with 19.9% having one type of DM complication and 24.6% having multiple complications. A proportion of 55.5% of diabetic patients had no DM complications, as shown in Fig 2. The prevalence of DM complications was higher in type 2 DM at 43.9% compared to 20.0% in type 1 DM. Among 7952 DM patients, 6.3% had retinopathy, 8.2% had nephropathy, 13.3% had peripheral circulatory complications, 57.8% had neuropathy, and 14.5% had other specified complications (Fig 3).

### Predictors of poor glycaemic control

The proportion of poor glycaemic control is higher in DM patients with a disease duration of more than 7 years, in overweight individuals (63.5%), in those who do not practice self-blood glucose monitoring, in NHIF DM patients (67.8%), in insulin users (78.0%), and in DM patients using medications that raise blood glucose levels (64.7%). The results showed that older age, a duration of DM of more than 10 years, insulin therapy, and a lack of awareness of glycaemic target goals were factors associated with poor glycaemic control, as shown in Table 3.

**Table 3:** Predictors for poor glycaemic control (n = 324).

Variables	Adjusted OR; 95%CI	P-Value
Young age	1	0.03
Old age	2.46 (1.28–6.01)	
Duration of disease	3.15 (2.22–6.55)	0.016
Insulin therapy	3.07 (2.10–6.12)	0.022
Self-glucose monitoring	3.42 (2.17–5.97)	0.001

### Discussion

#### Prevalence of poor glycaemic control among DM patients

In this study, it was observed that 66.4% of DM patients attending BMC had poor glycaemic control. These results are approximately similar to a study conducted in Dar-es-Salaam, Tanzania, which reported that 66.1% of DM patients had poor glycaemic control [5]. This finding is also similar to a meta-analysis study conducted in Ethiopia, which reported the proportion of poor glycaemic control to be 61.9% [7]. The prevalence of poor glycaemic control (66.4%) was lower compared to a study conducted in Saudi Arabia, which reported a prevalence of 74.9% [8], and in Iraq, which reported a prevalence of 86.2% [9]. These variations could be due to differences in the method of glucose measurements, cut off points, lifestyle, genetics, and environmental factors.

### Prevalence of diabetic complications among DM patients

Diabetic complications were found in 44.5% of diabetic patients attending BMC based on patient records reviewed over one year, which is lower compared to a study conducted in Kilimanjaro, Tanzania, that reported an overall prevalence of 50.2% [10]. This difference could be attributed to poor lifestyle factors including alcohol consumption. A study by Joel *et al*. revealed that Kilimanjaro region had a higher rate of alcohol consumption compared to the Mwanza region [11]. It is well-established that alcohol consumption is among the predictors of poor glycaemic control among DM patients [12].

Micro vascular complications were prevalent in 32.3% of diabetic patients, a finding similar to a study conducted in Kenya, which reported a prevalence of 35.3% [13]. This study shows that a higher proportion, 57.8% of DM patients, had neurological complications, mainly neuropathy and stroke. A study conducted in Kilimanjaro, Tanzania, reported a higher prevalence of peripheral neuropathy at 72.2% [14], These variations observed are likely due to differences in lifestyle among DM patients in both regions [11].

## Predictors associated with poor glycaemic control

The study showed that older participants with diabetes, specifically in the age category of 55–64 years, tended to have poorer glycaemic control compared to their younger counterparts. Studies conducted in Tanzania and Ethiopia reported that the age group of 40–59 had a higher proportion of poor glycaemic control [15]. Another study done in Ethiopia showed that younger populations were associated with poor glycaemic control [3]. The observed variations could be due to differences in population characteristics and age distribution among different studies.

Patients with DM for more than ten years were found to be 3.25 times more likely to have poor glycaemic control than those with shorter durations. This finding is similar to a study conducted in Ethiopia [16]. The reason for poor glycaemic control in patients with a longer duration of diabetes is attributed to a decline in pancreatic function over time, leading to lower levels of insulin secretion and increased insulin resistance [17].

Patients on insulin therapy had 3.08 times higher odds of poor glycaemic control compared to those on different treatment regimens. This finding is similar to studies conducted in Ethiopia and Yemen [18,19]. The reasons for poor glycaemic control among insulin users could be attributed to a lack of knowledge on insulin storage and the proper use of disposable syringe-needles. Finally, patients without established glycaemic target goals were found to be 3.45 times more likely to have poor glycaemic control than those who had established goals.

## Study limitations

This study included diabetic patients attending BMC and therefore cannot be generalized to the entire national population. Additionally, the cross-sectional nature of the study does not establish a cause-and-effect relationship between the independent variables and the outcome variable, thus preventing confirmation of causality. The study could also have overestimated the prevalence of poor glycaemic control hence needs to be interpreted with care because we did not include newly diagnosed DM patients because we thought having experience in insulin administration could help in controlling blood glucose.

## Policy implications

To address the challenges highlighted in the study, it is crucial to implement a multifaceted approach aimed at improving glycaemic control and reducing the prevalence of diabetic complications. This includes targeted educational campaigns to increase awareness among diabetic patients, particularly regarding the importance of glycaemic control and adherence to treatment regimens. Regular monitoring and screening for complications, especially neuropathy, should be emphasized, alongside individualized treatment plans tailored to patient needs. Collaboration among healthcare professionals in multidisciplinary teams can enhance the provision of comprehensive care while promoting healthy lifestyle interventions can further support long-term management of diabetes. By integrating these strategies, healthcare systems can strive to achieve better outcomes and enhance the overall well-being of diabetic patients.

## Conclusion

A higher proportion of diabetic patients were unable to achieve good glycaemic control, indicating a need for greater efforts to improve glycaemic control. About two-fifths of the study participants experienced diabetic complications, with diabetic neuropathy being the most common complication. Older age, a duration of DM of more than 10 years, insulin therapy, and lack of awareness of glycaemic target goals were significantly associated with poor glycaemic control.

## Supporting information

S1 File Extracted data from medical files (April 2022 ‐ March 2023). (DOCX)
