# Supplementary material for: Factors associated with glycaemic control and diabetes complications in patients at Bugando Medical Centre, Mwanza, Tanzania: A cross-sectional study design
Source: PLoS One. 2024 Aug 30;19(8):e0308659. doi: 10.1371/journal.pone.0308659 (PMC11364410; doi:10.1371/journal.pone.0308659)
Supplement: S1 File — (DOCX) [file pone.0308659.s001.docx]

Supporting Information files

**Table 1. Extracted data of the medical files from hospital management system from April 2022 up to March 2023**

|  | Type 1 DM | Type 2 DM | Renal complications | | Ophthalmic complications | | Peripheral circulatory complications | | Neurological complications | | Multiple complications | | Other specified complications | |
| --- | --- | --- | --- | --- | --- | --- | --- | --- | --- | --- | --- | --- | --- | --- |
|  |  |  | Type 1 DM | Type 2 DM | Type 1 DM | Type 2 DM | Type 1 DM | Type 2 DM | Type 1 DM | Type 2 DM | Type 1 DM | Type 2 DM | Type 1 DM | Type 2 DM |
| Male | 228 | 2864 | 2 | 65 | 2 | 44 | 6 | 113 | 19 | 368 | 11 | 893 | 8 | 97 |
| Female | 281 | 4579 | 7 | 56 | 8 | 45 | 1 | 89 | 14 | 513 | 13 | 1024 | 11 | 114 |
| Subtotal | 509 | 7443 | 9 | 121 | 10 | 89 | 7 | 202 | 33 | 881 | 24 | 1935 | 19 | 211 |
| Total | 7952 | | 130 | | 99 | | 209 | | 914 | | 1959 | | 230 | |
